# Dietary legume consumption reduces risk of colorectal cancer: evidence from a meta-analysis of cohort studies

**DOI:** 10.1038/srep08797

**Published:** 2015-03-05

**Authors:** Beibei Zhu, Yu Sun, Lu Qi, Rong Zhong, Xiaoping Miao

**Affiliations:** 1 State Key Laboratory of Environment Health (Incubation), MOE (Ministry of Education) Key Laboratory of Environment & Health, Ministry of Environmental Protection Key Laboratory of Environment and Health (Wuhan), and Department of Epidemiology and Biostatistics, School of Public Health, Tongji Medical College, Huazhong University of Science and Technology, Wuhan, China; 2 Department of Nutrition, Harvard School of Public Health, Boston, USA

## Abstract

Previous epidemiological studies on the relation between dietary legume consumption and risk of colorectal cancer (CRC) remain controversial. We conducted a meta-analysis based on prospective cohort studies to investigate the association between dietary legume consumption and risk of CRC. Fourteen cohort studies were finally included, containing a total of 1903459 participants and 12261 cases who contributed 11628960 person-years. We found that higher legume consumption was associated with a decreased risk of CRC (RR, relative risk = 0.91; 95% CI, confidence interval = 0.84–0.98). Subgroup analyses suggested that higher legume consumption was inversely associated with CRC risk in Asian (RR = 0.82; 95% CI = 0.74–0.91) and soybean intake was associated with a decreased risk of CRC (RR = 0.85; 95% CI = 0.73–0.99). Findings from our meta-analysis supported an association between higher intake of legume and a reduced risk of CRC. Further studies controlled for appropriate confounders are warranted to validate the associations.

CRC is the third most commonly diagnosed cancer in males and the second in females^1^. Over the past few decades, CRC incidence has been rapidly increasing, especially in developed countries^2^. The considerable geographic variation in incidence of CRC suggests that life style, especially dietary factors, may play vital roles in the development of CRC^3^,^4^,^5^. Various dietary factors have been related to the etiology of colorectal cancer, however, so far only the effects of alcohol and consumption of processed and red meat have been established^6^,^7^,^8^,^9^,^10^,^11^.

Legumes are a diverse group of foods, including soybeans, peas, beans, lentils, peanuts, and other podded plants, which are widely cultivated and consumed. Soybeans are unique among the legumes because they are a concentrated source of isoflavones, which are structurally similar to endogenous estrogen and can bind to estrogen receptors. Previous studies suggested isoflavones might impact cancer initiation and progression through estrogenic and antiestrogenic activities^12^. Besides isoflavones, legumes are good sources of dietary protein, vitamin E, vitamin B, selenium, and lignans, which may also have potential cancer-preventive effects^13^.

Despite such biological fitness^14^, epidemiological studies investigating the association between legumes intake and risk of CRC generated conflicting results. Recently, a meta-analysis of four cohort and seven case-control studies found that consumption of soy foods might be associated with a reduced risk of CRC risk among women but not among men^15^, however, case-control studies are prone to recall and selection bias. Another more recent meta-analysis of cohort studies did not find significant association between intake of legume fiber and CRC^16^. This study merely focused on the legume fiber and only four cohort studies were finally included, and might not have sufficient power to detect modest associations. Therefore, we conduct a meta-analysis of currently available prospective cohort studies and assessed all kinds of legume foods, with aims to reach a consistent conclusion regarding association s between higher legume consumption and CRC risk.

## Results

### Study characteristics

**Figure 1:**
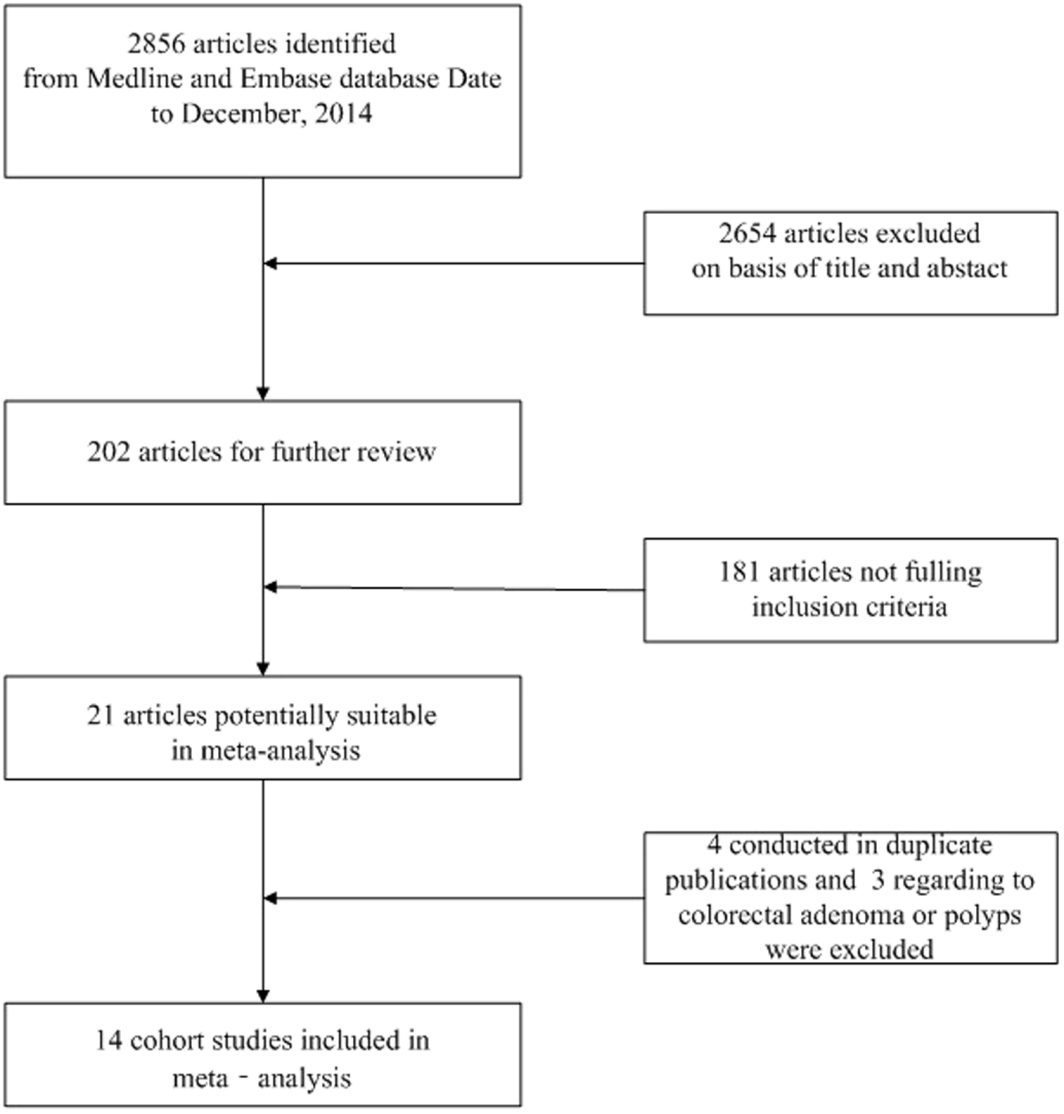
The flow chart of search and selection.

We identified 21 potentially relevant full text publications^17^,^18^,^19^,^20^,^21^,^22^,^23^,^24^,^25^,^26^,^27^,^28^,^29^,^30^,^31^,^32^,^33^,^34^,^35^,^36^,^37^. Four conducted in duplicate publications^24^,^26^,^34^,^36^ and three regarding to colorectal adenoma or polyps^17^,^18^,^19^ were excluded. Thus, fourteen cohort studies^20^,^21^,^22^,^23^,^25^,^27^,^28^,^29^,^30^,^31^,^32^,^33^,^35^,^37^ were included in the meta-analysis, containing a total of 1903459 participants and 12261 CRC cases who contributed 11628960 person-years of follow-up. The flow chart of search and selection is presented in Figure 1. Food frequency questionnaire was used for dietary assessment in all of these studies. Seven of the fourteen studies involved US populations^25^,^28^,^29^,^31^,^32^,^35^,^37^, five were from Asia^20^,^21^,^22^,^27^,^33^, three were from Japan^21^,^22^,^27^, two were from China 20,33, and the other two were from Europe 23,30. Of the fourteen studies analyzed, nine provided data on women^20^,^21^,^22^,^25^,^28^,^30^,^31^,^32^,^35^ and six on men^21^,^22^,^28^,^30^,^32^,^33^, only five studies presented separate data for men and women^21^,^22^,^28^,^30^,^32^, one study provided data for men only 33 and four was conducted with women only^20^,^25^,^31^,^35^. Most studies provided relative risk estimates adjusted for smoking (n = 11), BMI (n = 10), red or processed meat (n = 10) and family history of CRC (n = 9), a few studies adjusted for fruit or vegetable (n = 3). Only five studies found a statistically significant inverse relationship between legume intake and CRC risk^20^,^21^,^25^,^27^,^35^. More detailed characteristics of the included studies are summarized in Table 1.

### Overall association between legume intake and CRC risk

**Figure 2:**
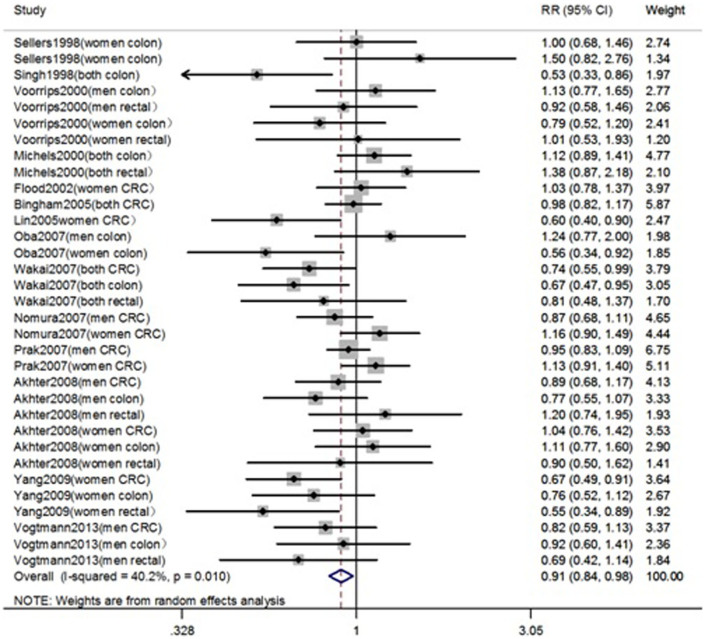
Forest plot of legumes consumption and risk of colorectal cancer.

Fourteen cohort studies were included in the analysis of the highest versus lowest intake of legume and risk of colorectal cancer. The summary relative risk was 0.91 (95% CI = 0.84–0.98; *P* = 0.01) and test of heterogeneity *I*^2^ = 40.2% (*P* = 0.01) (Figure 2), indicating an inverse association between legume intake and CRC risk.

### Meta-regression

We conducted a meta-regression to comprehensively explore the source of heterogeneity. Eleven factors such as country, gender, cancer site, study size, follow-up period, number of cases, whether adjusted factors such as energy, BMI, smoking, fruit, red/processed meat. were included in the meta-regression model. In this model, the Adj R-squared was 100.00%, and Prob > F was 0.02, which indicated that the model was significant. After 100 times permutation, legume species, follow-up duration and whether controlled for red/processed meat intake appeared to be significant to explain the between-study heterogeneity.

### Subgroup analyses

To identity underlying sources of heterogeneity among these studies, we performed subgroup analyses. In subgroup analyses defined by population, gender, cancer type, participants, number of cases and duration of follow-up, dietary legume consumption was not significantly associated with risk of CRC in most subgroups, excepted in Asia (RR = 0.82, 95% CI = 0.74–0.91) (Table 2). We further carried out the subgroup analyses according to adjustment, in the subgroups of studies that adjusted for age, body mass index, red or processed meat, inverse associations were significant. The RRs were 0.88 (95% CI = 0.81–0.96), 0.86 (95% CI = 0.78–0.95), and 0.89 (95% CI = 0.81–0.98) for analyses adjusting for age, BMI, red or processed meat, respectively. More detailed results of the subgroup analyses are summarized in Table 2.

### Legume species

Stratified according to legume species, we found an inverse association between soybeans intake and CRC risk (RR = 0.85, 95% CI = 0.73–0.99). Legume fiber intake marginally associated with a decreased risk of CRC (RR = 0.85, 95% CI = 0.72–1.00); however, we did not observe this inverse association in subgroup of beans (RR = 1.00, 95% CI = 0.89–1.13) (Table 3).

### Sensitivity analysis

When each study was excluded from the meta-analysis in turn, the pooled RRs did not change fundamentally, indicating that our results could not be solely attributed to the effect of a single study. The RR ranged from 0.89 (95% CI = 0.82–0.97) when the NIH-AAPR Diet and Health Study^36^ was excluded to 0.92 (95% CI = 0.85–0.99) when the Women's Health Study (WHS)^29^ was excluded.

### Publication bias

**Figure 3:**
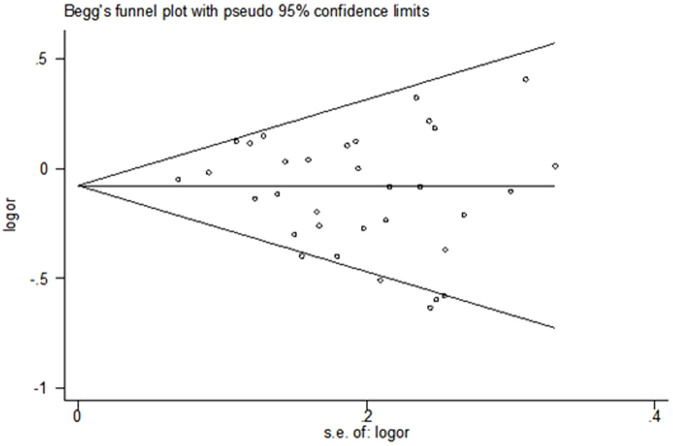
Funnel plot of publication bias.

The result of Egger's test (*P* = 0.16) or Begg's test (*P* = 0.31) indicated no evidence of substantial publication bias (Figure 3).

## Discussion

We systematically reviewed fourteen published prospective cohorts on the relationship between legume consumption and CRC incidence. Our meta-analysis supports an inverse association between higher intake of legume and risk of colorectal cancer. Among all the legume species, soybeans and legume fibers revealed to be associated with a decreased risk of CRC. Higher consumption of legume reduced the risk of CRC among Asians needs extra validation.

The mechanism underlying a possible protective effect of legume intake on CRC risk might be complex because of a great variety of anti-carcinogens in legumes. The most important anticancer composition of legume food is flavonoids, especially isoflavones. Flavonoids from legume food not only inhibit the growth of tumor cells, but also induce cell differentiation^38^. The inhibitory effects of flavonoids on the growth of malignant cells might be a consequence of their interference with the protein kinase activities involved in the regulation of cellular proliferation and apoptosis^39^. In addition, legumes are rich in dietary fiber, which may increase stool bulk, decrease transit time and dilute potential carcinogens in the gastrointestinal tract. Further, fiber from legume stimulates bacterial anaerobic fermentation which results in production of short-chain fatty acids, such as butyrate, which inhibits growth, induces apoptosis and cell cycle arrest, and promotes differentiation in CRC cells^40^. Furthermore, legumes are good sources of dietary protein, vitamin E, vitamin B, selenium, and lignans with potential cancer-preventive effects. Legumes have a high content of vitamin B6^41^ and vitamin B6 intake was reported to reduce risk of colorectal cancer^42^. In addition to its direct cancer preventive effects, legume intake may affect disease risk indirectly as well. For example, higher intake of legumes may replace other sources of protein in the diet such as meat^43^.

Based on the results of meta-regression analysis, we think legume species, follow-up duration and whether controlled for red/processed meat are the major source of between-study heterogeneity. In subgroup analyses, we found an inverse association between legume intake and CRC risk among Asian. Possible reason for this result is that dietary patterns containing higher levels of legumes in Asia population. Subgroups analyses according to legumes species revealed higher intake soybeans reduced risk of colorectal cancer. Soybeans are unique among the legumes because they are a concentrated source of isoflavones, such as genistein and daidzein, which may have cancer preventive properties. These compounds may compete with estrogens by binding to the estrogen receptor and thereby reduce cancer risk. More importantly, when stratified according to the confounders controlled, we found that combining those studies adjusted for BMI, vegetables and red meat intake revealed an inverse association between higher consumption of legume and risk of colorectal cancer. These three factors have been previously related to the risk of CRC^44^,^45^,^46^, and failure in adjustment for these factors might bias the associations. For the discrepancy in the subgroup analysis according to number of cases and duration of follow-up time, we think usually small sample size (<500) generate less stable results, so it is difficult to exclude the possibility that the positive association is due to chance. Referring to longer follow-up duration (≥10) lacked the significant association, we speculated that it might be due to small sample size without enough power to detect the association or because with longer follow-up time, the population might be older and other aging-related factors might contribute more to the incidence of cancer and therefore dilute the associations tested for the exposures tested.

We found legume fiber consumption is marginally associated with a decreased risk of colorectal cancer, which is inconsistent with a previous meta-analysis^16^. This discrepancy may be partly due to the larger sample size of our study than the others and exclusion of the studies without adjustment for the potential confounders. Regarding to gender, we did not find that legume consumption was associated with a reduced risk of CRC among women, but was marginally associated with a decreased risk of CRC among men, which is inconsistent with another previous meta-analysis^15^. The explanation for this disagreement might be that previous meta-analysis included both case-control and cohort studies.

Our meta-analysis has several strengths. First our current study is based on prospective cohort studies, which is unlikely to be influenced by recall bias and selection bias. Second, combining a large number of studies renders us sufficient power to detect potential modest associations. In addition, sensitivity analyses and publication bias indicated our findings were generally robust and reliable.

Several limitations of our study should also be acknowledged. First, we did not have sufficient data to conduct a dose-response meta-analysis, which made us unable to evaluate the precise relationship. Besides, it is possible that our results were affected by the unmeasured or residual confounding by other dietary or lifestyle factors. Furthermore, because these studies conducted in different countries and populations, the items they measured legume consumption varied. So our findings may be influenced by the misclassification of legume consumption and the inability of providing accurate measurement of intake also limited the impact of our study. In summary, our meta-analysis suggests that a higher intake of legume is associated with a reduced risk of colorectal cancer. Further studies with better dietary assessment tools and adjustment for appropriate confounding factors are warranted to confirm the associations.

## Methods

### Identification of studies

To get all the eligible studies relating to the legume consumption and risk of colorectal cancer, we conducted a systemic retrieval through Medline and Embase databases date to December 2014. We used the following terms as key words in combination for the literature search: legume, soy, beans, peas, soybeans, tofu, soymilk, vegetable, diet and colorectal cancer, restricted to English. In addition, reference lists of retrieved articles and current review articles were scanned manually for all relevant additional studies. When multiple studies pertained to the same or partially overlapping population, we used the results with the longest follow-up time or largest sample size.

### Inclusion criteria

We systematically examined the identified studies, studies met the following criterion were included: 1) a prospective cohort design; 2) the exposure was legume consumption, including tofu or soybeans, peas, beans, lentils, and other podded plants and all products made of them; 3) the outcome was risk of colorectal cancer, incidence of colorectal cancer; 4) provided or allowed calculation of RR with 95% CI. Studies were excluded if they 1) had a retrospective design; 2) were Non-human, in vitro research, case reports; 3) focused on the recurrence, growth; 4) focused on adenoma; and 5) did not adjust for confounders.

### Data extraction

All data were extracted independently and cross-checked by two authors (YS and BBZ). For the eligible studies, the following data were extracted: first author, year of publication, geographic region, study name, follow-up period, number of participants/person-years of follow-up, number of cases, demographics of participants, cancer sites, species and amount of legumes consumption, relative risks and 95% CI for the highest versus the lowest intake, and adjustment for confounders in the analysis. Any results stratified by sex or tumor site were treated as separate reports.

### Statistical analysis

We extracted the maximally adjusted RR (95% CI) in order to control for confounding factors. We quantified the relationship between legumes consumption and CRC risk by pooling the RRs for the highest category compared with the lowest category. Q statistic test was applied to assess between-study heterogeneity^47^ and the degree of heterogeneity was further quantified using the I2 statistic^48^. *I*^2^ values of 25, 50, and 75% corresponded to low, moderate, and high degrees of heterogeneity, respectively^48^. Statistically significant heterogeneity was considered when *P* < 0.05. We pooled the RRs in a random effects model described by DerSimonian and Laird used^49^, which takes into account both within- and between-study variability. We conducted a meta-regression to comprehensively explore the source of heterogeneity. Eleven factors such as country, gender, cancer site, study size, follow-up period, number of cases, whether adjusted factors such as energy, BMI, smoking, fruit, red/processed meat. were included in the meta-regression model. Subgroup analyses were further performed, if feasible, according to legume species, sex and site, geographic region, number of cases and duration of follow-up and confounders adjusted for. Sensitivity analyses were conducted by excluding each study in turn to evaluate the stability of the results. Publication bias was assessed using the funnel plot and Egger's test. Any asymmetry observed or P < 0.05 indicated potential publication bias. All analyses were performed with comprehensive meta-analysis^50^ and were carried out by Stata version 10.0 (STATA Corp, College Station, TX).

## Author Contributions

Conceived and designed the study strategy: X.P.M.; Acquisition of data: statistical analysis and interpretation of data B.B.Z. and Y.S.; Drafting or revision of the manuscript: B.B.Z. and Y.S.; Reference collection and data management: Y.S.; Wrote the manuscript: B.B.Z., Y.S. and L.Q.; Prepared the tables and figures: Y.S. and R.Z.; Study supervision: X.P.M.; All authors reviewed the manuscript.

## Figures and Tables

**Table 1:** Characteristics of included studies of the association between legume intake and CRC risk

Study	Study name	Country	Gender	Study period	Age	No. of cases	No. of participants	Adjustment
Sellers 1998 (39)	Iowa Women's Health Study	USA	Female	1986–1995	55–69	241	41837	Age at baseline, total energy intake, and history of rectal colon polyps
Singh 1998 (33)	Adventist Health Study	USA	Both	1976–1982	≥25	157	34198	Age at baseline, sex, BMI, physical activity, parental history of colon cancer, current smoking, past smoking, alcohol consumption, and aspirin use
Voorrips 2000 (34)	Netherlands Cohort Study	Netherland	Both	1986–1992	55–69	910	120852	Age, family history of colorectal caner, and category of alcohol intake
Michels 2000 (41)	Nurses' Health Study (NHS) and Health Professionals' Follow-up Study (HPFS)	USA	Both	1980–1996	Women30–55Men40–75	1181	136089	Age, family history of colorectal cancer, sigmoidoscopy, height, BMI, pack-years of smoking, alcohol intake, physical activity, menopausal status(women), postmenopausal hormone use(women), aspirin use, vitamin supplement intake, total caloric intake, and red meat consumption
Flood 2002 (35)	Breast Cancer Detection Demonstration Project (BCDDP)	USA	Female	1987–1989, 1993–1998	Mean62	485	45490	Multivitamin supplement use, BMI, height, use of nonsteroidal antiinflammatory drugs, smoking status, education level, physical activity, and intakes of fruit, grains, red meat, calcium, vitamin D, and alcohol
Bingham 2005 (27)	European Prospective Investigation into Cancer and Nutrition (EPIC)	Europe	Both	1992–2004	25–70	1721	519978	Age, sex, energy from nonfat sources, energy from fat sources, height and weight, and stratified for center, folate and physical activity, alcohol consumption, smoking status, educational level, and intake of meat and processed meat
Lin 2005 (29)	Women's Health Study (WHS)	USA	Female	1993–2003	≥45	223	39876	Age, randomized treatment assignment, BMI, family history of CRCin a first degree relative, history of colon polyps, physical activity, smoking status, baseline aspirin use, red meat intake, alcohol consumption, total energy intake, menopausal status and baseline post-menopausal HT use, folate intake and multivitamin use
Oba 2007 (25)	Takayama study	Japan	Both	1992–2000	≥35	213	30221	Age, height, alcohol intake, smoking status, BMI, physical exercise, coffee intake, and use of hormone replacement therapy (women only)
Wakai 2007 (31)	Japan Collaborative Cohort Study (JACC)	Japan	Both	1988–1997	40–79	443	43115	Age, sex, area, educational level, family history of CRCin parents and/or siblings, alcohol consumption, smoking, BMI, daily walking habits, exercise, sedentary work, consumption of beef and pork, energy intake, and energy-adjusted intakes of folate, calcium, and vitamin D
Nomura 2007 (32)	Multiethnic cohort study (MEC)	USA	Both	1993–2001	45–75	2110	191011	Age, family history of colorectal cancer, history of colorectal polyp, pack-years of cigarette smoking, BMI, hours of vigorous activity, aspirin use, multivitamin use, and replacement hormone use (women), alcohol, red meat, folate, vitamin D, and calcium
Park 2007 (36)	NIH-AAPR Diet and Health Study	USA	Both	1995–2000	50–71	2972	488043	Education, physical activity, smoking, alcohol consumption, and intake of red meat, dietary calcium, and total energy
Akhter 2008 (26)	Japan PublicHealth Center(JPHC)	Japan	Both	1998–2004	45–74	886	83063	Age, public health center area, history of diabetes mellitus, BMI, leisure time physical activity, cigarette smoking, alcohol drinking, and intake of vitamin D, dairy products, meat, vegetable, fruit, and fish, menopausal status and current use of female hormones(women)
Yang 2009 (24)	Shanghai Women's Health Study	China	Women	1997–2005	40–70	321	68412	Age, education, household income, physical activity, BMI, menopausal status, family history of colorectal cancer, total calorie intake, and average intakes of fruit, vegetables, red meat, nonsoy calcium, nonsoy fiber, and nonsoy folic acid
Vogtmann 2013 (37)	Shanghai Men's Health Study (SMHS)	China	Male	2002–2010	40–74	398	61274	Age, total energy intake, red meat intake, total meat intake, education, income, occupation, smoking status, alcohol consumption, BMI, MET hours of exercise participation, history of diabetes mellitus, and family history of colorectal cancer

Abbrevation: BMI, body mass index; HT, hormone therapy; MET, metabolic equivalent.

**Table 2:** Results of subgroup analyses

Factor	No. of studies	RR (95%CI)	*P* for test	Heterogeneity	
				*I*^2^ (%)	*P*
All	14	0.91(0.84–0.98)	0.01	40.2	0.01
**Population**					
USA	7	0.99(0.87–1.13)	0.88	53.7	0.02
Asian	5	0.82(0.74–0.91)	<0.01	19.5	0.23
Europe	2	0.97(0.84–1.12)	0.68	0.0	0.80
**Gender**					
Male	6	0.92(0.85–1.01)	0.07	0.0	0.71
Female	9	0.90(0.78–1.03)	0.13	52.5	0.01
**Cancer site**					
Colon cancer	9	0.89(0.77–1.04)	0.14	49.1	0.02
Rectal cancer	6	0.90(0.73–1.12)	0.34	29.9	0.19
**Study size**					
>100000	5	1.01(0.94–1.09)	0.78	0.0	0.54
<100000	9	0.83(0.75–0.92)	0.00	35.4	0.05
**Follow-up period**					
≥10	3	1.04(0.79–1.38)	0.77	60.6	0.04
<10	11	0.89(0.82–0.96)	<0.01	33.9	0.04
**No. of cases**					
<500	8	0.78(0.69–0.88)	<0.01	37.3	0.07
500–1500	3	1.00(0.90–1.12)	0.94	0.0	0.65
>1500	3	1.00(0.91–1.09)	0.94	9.7	0.35
**Age**					
Yes	12	0.88(0.81–0.96)	0.01	40.4	0.01
No	2	1.00(0.90–1.12)	0.96	0.0	0.40
**BMI**					
Yes	10	0.86(0.78–0.95)	<0.01	47.0	0.01
No	4	1.00(0.91–1.09)	0.93	0.0	0.73
**Energy**					
Yes	6	0.94(0.84–1.06)	0.32	33.4	0.15
No	8	0.89(0.81–0.98)	0.02	43.2	0.01
**Fruit, vegetable**					
Yes	3	0.88(0.76–1.00)	0.06	32.3	0.15
No	11	0.92(0.84–1.01)	0.08	43.7	0.01
**Red, processed meat**					
Yes	10	0.89(0.81–0.98)	0.02	51.1	0.01
No	4	0.93(0.82–1.06)	0.28	25.1	0.18

Abbreviation: RR, relative risk; CI, confidence interval.

**Table 3:** Stratified analysis according to legume species

Legume species	No. of studies	RR (95%CI)	*P* for test	Heterogeneity	
				*I*^2^ (%)	*P*
Beans	5	1.00 (0.89–1.13)	0.97	31.4	0.16
Soybeans	3	0.85 (0.73–0.99)	0.04	41.0	0.08
Legume fiber	4	0.85 (0.72–1.00)	0.05	54.1	0.04

Abbreviation: RR, relative risk; CI, confidence interval.
